# Histogram analysis based on DTI and NODDI for differentiating atypical high-grade glioma from primary central nervous system lymphoma

**DOI:** 10.3389/fneur.2025.1577811

**Published:** 2025-07-23

**Authors:** Shanshan Zhao, Xiaoyue Ma, Linlin Li, Eryuan Gao, Kai Zhao, Mengzhu Wang, Guang Yang, Hongbin Zheng, Jingliang Cheng, Guohua Zhao

**Affiliations:** ^1^Department of Magnetic Resonance Imaging, The First Affiliated Hospital of Zhengzhou University, Zhengzhou, China; ^2^Tianjian Laboratory of Advanced Biomedical Sciences, Zhengzhou, China; ^3^Dengfeng Hospital of Traditional Chinese Medicine, Dengfeng, China; ^4^MR Research Collaboration, Siemens Healthineers Ltd., Beijing, China; ^5^Shanghai Key Laboratory of Magnetic Resonance, East China Normal University, Shanghai, China; ^6^The Affiliated Hospital of Youjiang Medical University for Nationalities, Baise, China

**Keywords:** magnetic resonance imaging, neurite orientation dispersion and density imaging, histogram analysis, high-grade glioma, primary central nervous system lymphoma

## Abstract

**Background and purpose:**

Distinguishing between high-grade glioma (HGG) and primary central nervous system lymphoma (PCNSL) is of paramount clinical importance, as these entities necessitate substantially different therapeutic approaches. The differential diagnosis becomes particularly challenging when HGG presents without characteristic magnetic resonance imaging (MRI) features, making it difficult to differentiate from PCNSL. The diffusion tensor imaging (DTI) and neurite orientation dispersion and density imaging (NODDI) offer quantitative assessments of water molecule diffusion within tissues, thereby providing potential means to characterize microstructural differences between HGG and PCNSL. This study aims to evaluate the diagnostic efficacy of histogram analysis based on DTI and NODDI parameters in differentiating atypical HGG from PCNSL.

**Materials and methods:**

We retrospectively reviewed patients who underwent multi-*b*-value diffusion-weighted imaging (DWI) at our institution. The multi-*b*-value DWI was performed using a single-shot echo-planar imaging (EPI) sequence with six *b*-values (0, 500, 1,000, 1,500, 2,000, and 2,500 s/mm^2^) distributed across 30 directions. The DTI and NODDI model were employed to derive the parametric maps of apparent diffusion coefficient (ADC), fractional anisotropy (FA), intracellular volume fraction (ICVF), isotropic volume fraction (ISOVF), and orientation dispersion index (ODI). Two regions of interest (ROIs) were manually delineated within the enhancing tumor area and the peritumoral edema. Histogram features were extracted from these ROIs. Comparisons between HGG and PCNSL were performed. Receiver operating characteristic (ROC) curves were drawn, and the area under the curve (AUC), sensitivity, specificity, and accuracy were calculated. *p* < 0.05 was considered statistically significant.

**Results:**

A total of 55 patients (30 with atypical HGG and 25 with PCNSL), were included in this study. Several histogram features of parameters could be used to classify the HGG and PCNSL (*p* < 0.05). The 75th percentile of the orientation dispersion index (ODI_75th_) within the enhancing tumor region demonstrated the highest diagnostic performance (AUC = 0.985). At an optimal threshold of 0.604, ODI_75th_ yielded a sensitivity of 96%, a specificity of 93.33%, and an accuracy of 94.55% for distinguishing HGG from PCNSL.

**Conclusion:**

DTI-and NODDI-based histogram analysis demonstrates the potential to differentiate between atypical HGG and PCNSL. ODI_75th_ within the enhancing tumor region showed the most favorable diagnostic performance.

## 1 Introduction

Glioma and primary central nervous system lymphoma (PCNSL) are two common malignant primary brain tumors (1). Precise diagnosis is of great significance as the therapeutic strategies differ. PCNSLs are not recommended for surgery but rather receive chemotherapy, with or without irradiation, after biopsy. Meanwhile, HGGs necessitate surgical resection followed by concurrent chemoradiation (2–4). Magnetic resonance imaging (MRI) acts as the standard approach for brain tumor diagnosis. Most primary central nervous system lymphomas (PCNSLs) typically appear as homogeneously hyperintense lesions on T2-weighted images and rarely exhibit central necrosis. In contrast, high-grade gliomas (HGGs) are often associated with imaging features such as central necrosis, hemorrhage, or ring-like enhancement, which generally facilitate their diagnosis (5) However, it has been reported that approximately 22.2% of HGGs do not show obvious intra-tumoral necrosis (6), and about 4.4% of PCNSLs may present with ring enhancement, potentially complicating differential diagnosis in atypical cases (7). Distinguishing HGG from PCNSL can be challenging when conventional MRI features are atypical. To address this challenge, researchers are actively exploring advanced imaging analysis techniques (8) and functional MRI (9). Diffusion MRI can provide information on the tissue microenvironment and help in diagnosis. The single-exponential model diffusion-weighted imaging (DWI), diffusion tensor imaging (DTI), and diffusion kurtosis imaging (DKI) have been utilized to differentiate HGG from PNCSL (4, 10–12). Advanced diffusion models have shown promising clinical applications. Recently, the neurite orientation dispersion and density imaging (NODDI) model (13) has garnered attention among radiologists (14). NODDI has been applied in glioma grading (15), genotyping (16), and differential diagnosis (17–19). In this research, we investigate the application of DTI-and NODDI-based histogram analysis for differentiating atypical HGG from PCNSL.

## 2 Materials and methods

This retrospective study was approved by the Scientific Research and Clinical Trial Ethics Committee of the First Affiliated Hospital of Zhengzhou University, and informed consent was waived due to the purely retrospective analysis (Approval Number: 2019-KY-231).

### 2.1 Study participants

A total of 210 patients were included in this study between September 2018 and October 2022, who underwent MRI including routine sequences and multi-*b*-value diffusion-weighted imaging (DWI), and were pathologically diagnosed with either grade 3–4 glioma (20) or PCNSL according the 2021 World Health Organization (WHO) classification of central nervous system tumors at our hospital. A total of 155 patients were excluded for: (1) anti-tumor treatment or biopsy prior to MRI scanning (*n* = 3); (2) surgery or biopsy not performed within 2 weeks of the MRI examination (*n* = 6); (3) images reviewed by two radiologists (SZ with 9 years of experience and XM with 11 years of experience) with severe motion or susceptibility artifacts (*n* = 2); (4) lesions with more than 13% necrosis in the enhancing tumor (11) (*n* = 144). Finally, 30 patients with atypical HGG and 25 with PCNSL were recruited.

### 2.2 MRI protocol

All patients were scanned using a 3.0 T MRI scanner (MAGNETOM Prisma; Siemens Healthineers, Erlangen, Germany) with a 64-channel head and neck integrated coil. The imaging sequences included: (1) axial T2 dark-fluid: TR/TE, 8,000/81 ms; FOV, 220 × 220 mm^2^; acquisition matrix, 314 × 314; slice thickness, 5.0 mm; (2) axial multi-*b*-value DWI: single-shot echo-planar imaging (EPI) sequence, with five non-zero *b*-values (500, 1,000, 1,500, 2,000, and 2,500 s/mm^2^) distributed across 30 directions, and one zero *b*-value (*b* = 0 s/mm^2^); TR/TE, 2,500/71 ms; FOV, 220 × 220 mm^2^; acquisition matrix, 100 × 100; slice thickness, 2.2 mm; and 3. 3D contrast-enhanced T1 magnetization-prepared rapid gradient echo (CE-T1 MPRAGE): TR/TE, 2300/2.32 ms; FOV, 240 × 240 mm^2^; acquisition matrix, 266 × 266; slice thickness, 0.9 mm. The acquisition time for the multi-*b*-value DWI sequence was 6 min and 34 s. The T1 MPRAGE sequence was performed following the administration of 0.2 mol/kg body weight of gadopentetate dimeglumine (Magnevist, Bayer Schering Pharma AG, Berlin, Germany). All sequences covered the entire brain.

### 2.3 Image processing and analysis

**Figure 1:**
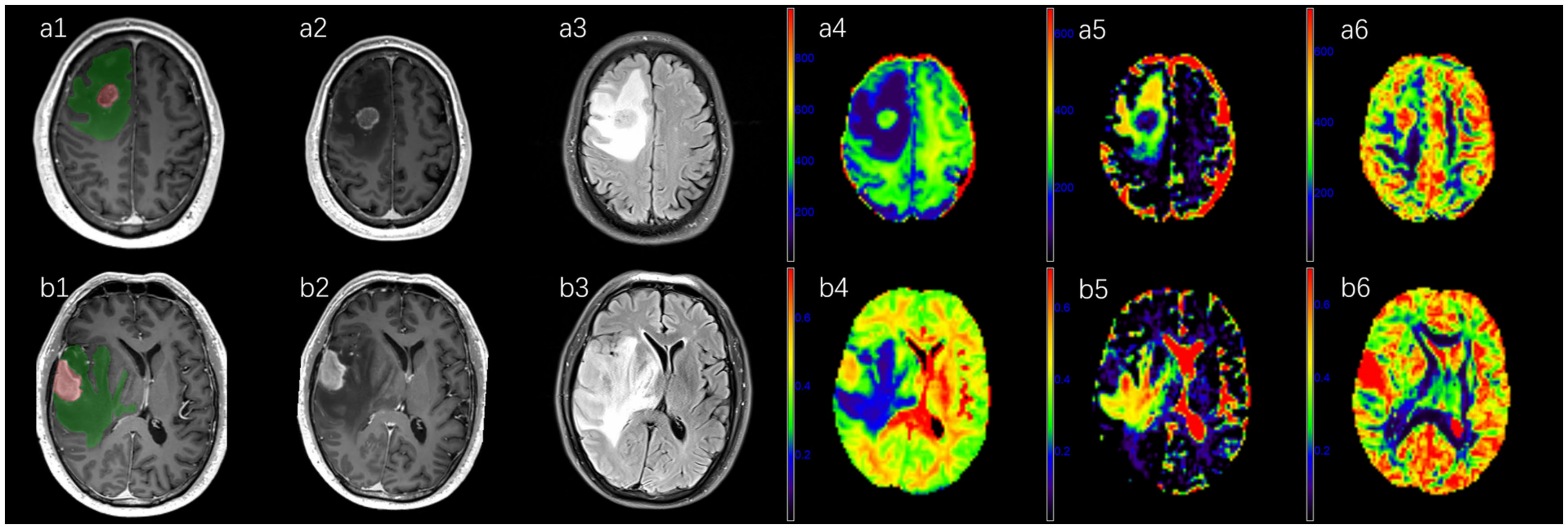
A 62-year-old male diagnosed with atypical high-grade glioma in the right frontal lobe **(a1–a6)** and a 59-year-old male diagnosed with primary central nervous system lymphoma in the right temporal lobe **(b1–b6)**. CE-T1-MPRAGE **(a2,b2)** and T2-tirm-dark-fluid **(a3,b3)** images depict peritumoral edema in green and the enhancing area in red **(a1,b1)**. Parametric maps of NODDI **(a4–a6,b4–b6)** include intracellular volume fraction **(a4,b4)**, isotropic volume fraction **(a5,b5)** and orientation dispersion index **(a6,b6)**.

Diffusion Kit Eddy tool^1^ (21) was used to perform eddy current and motion correction for the multi-*b*-value DWI images. NeuDilab, a software based on DIPY^2^ (22), was used for post-processing for the multi-*b*-value DWI images. The apparent diffusion coefficient (ADC), fractional anisotropy (FA), intracellular volume fraction (ICVF), isotropic volume fraction (ISOVF), and orientation dispersion index (ODI) were calculated. CE-T1 MPRAGE images and all parametric maps were registered to the axial T2 dark-fluid images. Two regions of interest (ROIs) were manually delineated with reference to the CE-T1 MPRAGE and T2 dark-fluid images by two radiologists (SZ with 9 years of experience and XM with 11 years of experience), who were blinded to the pathological results. The enhancing tumor area (ROI 1) was outlined on the registered CE-T1 MPRAGE images across all slices (Figure 1), excluding cystic, necrotic, and hemorrhagic regions. Peritumoral edema (ROI 2) was defined as the area surrounding the enhancing tumor margin with high signal intensity on all slices of the T2 dark-fluid images (Figure 1). Registration and delineation were performed using ITK-SNAP software (version 3.8.0, http://www.itksnap.org) (23). The two ROIs and all parametric maps were imported into MATLAB (version. R2017b; MathWorks, Natick, MA, United States) to compute histogram features of parameters in each ROI, including minimum (min), mean, maximum (max), 10th percentile (10th), 25th percentile (25th), median, 75th percentile (75th), 90th percentile (90th), variance, skewness, and kurtosis.

### 2.4 Statistical analyses

Statistical analyses were conducted using SPSS software (version 21.0; SPSS Inc., Chicago, IL, United States). The normality of all histogram parameters was assessed using the Shapiro–Wilk test, and the homogeneity of variance was evaluated using Levene’s test. Data conforming to a normal distribution were presented as mean ± standard deviation, while non-normally distributed data were expressed as median (25th, 75th). Comparative analysis between the atypical HGG and PCNSL groups was performed using an independent *t*-test for normally distributed data with uniform variance, while the Mann–Whitney *U* test was used for the remaining datasets. Statistical significance was set at *p* < 0.05. Benjamini–Hochberg correction was applied to adjust the *p*-values of diffusion parameters for multiple comparisons. Receiver operating characteristic (ROC) analyses were conducted to evaluate the diagnostic efficiency of parameters showing significant differences. Areas under the curve (AUCs) were measured, and the corresponding sensitivity, specificity, and accuracy were calculated by selecting the cut-off value with the maximum Youden index.

## 3 Results

### 3.1 Patient demographics

Among the 55 patients, 30 were diagnosed with atypical HGG (18 males, 12 females; age range, 20–73 years; mean age, 51 ± 12 years), and 25 patients had PCNSL (14 males, 11 females; age range, 33–72 years; mean age, 59 ± 9 years). All cases were classified according to the 2021 WHO criteria. In the atypical HGG group, 26 patients were diagnosed with glioblastoma, IDH-wildtype (WHO grade 4), 2 with astrocytoma, IDH-mutant (WHO grade 3), and 2 with oligodendroglioma, IDH-mutant and 1p/19q-codeleted (WHO grade 3). All patients in the PCNSL group were diagnosed with large B-cell lymphoma.

### 3.2 Histogram parameter values in the enhancing area

**Figure 2:**
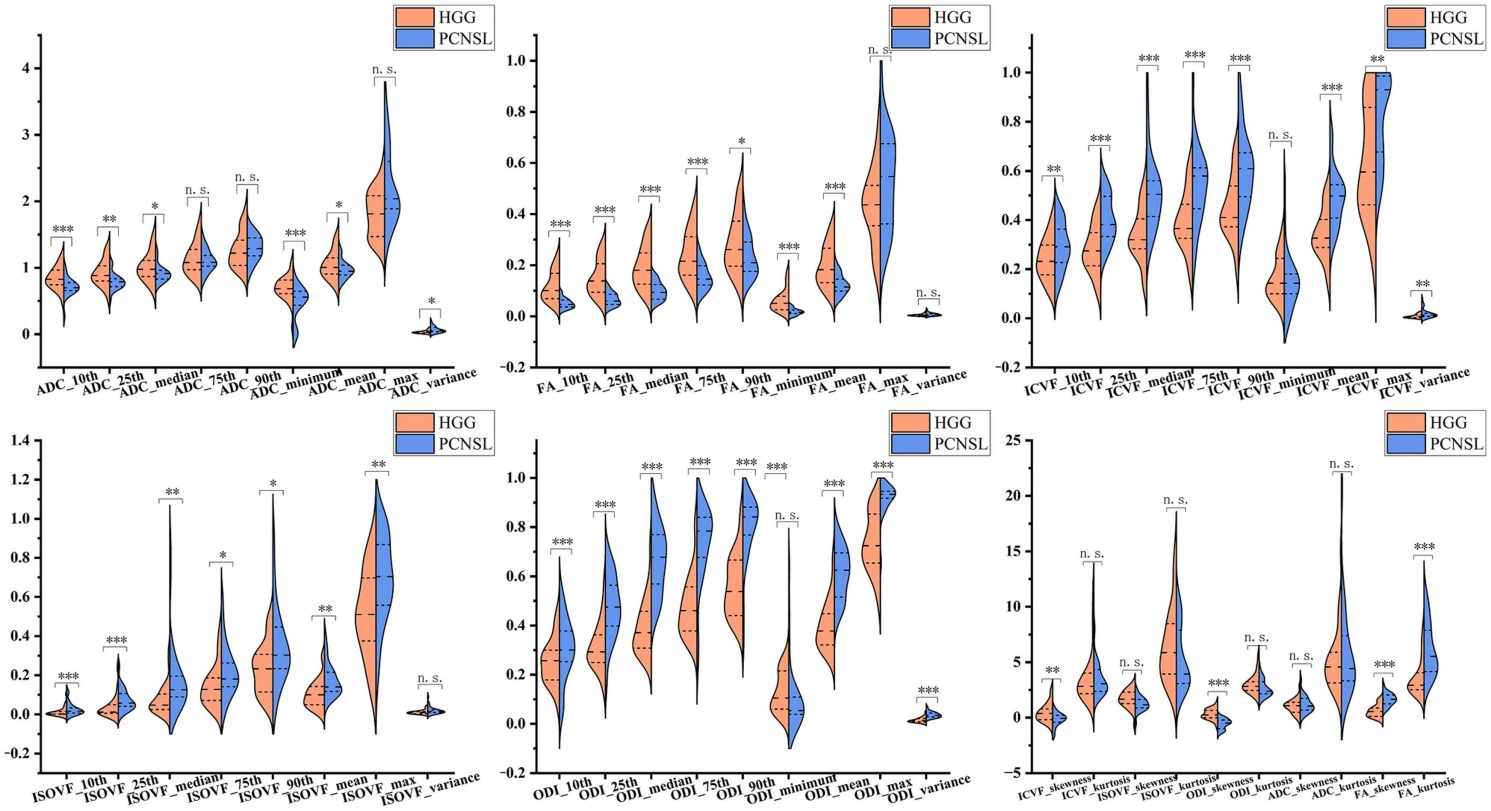
Violin graphs of histogram parameter values in the enhancing area. HGG, high-grade glioma; ADC, apparent diffusion coefficient; FA, fractional anisotropy; ICVF, intracellular volume fraction; ISOVF, isotropic volume fraction; ODI, orientation dispersion index; PCNSL, primary central nervous system lymphoma. ^*^0.01 < *p* ≤ 0.05; ^**^0.001 < *p* ≤ 0.01; ^***^p ≤ 0.001. n.s., no significance.

The histogram parameter (ADC, FA, ICVF, ISOVF, and ODI) in the enhancing area are shown in Table 1 and Figure 2. In the enhancing area, ADC_variance_, FA_kurtosis_, FA_skewness_, ICVF_mean_, ICVF_max_, ICVF_10th_, ICVF_25th_, ICVF_median_, ICVF_75th_, ICVF_90th_, ICVF_variance_, ISOVF_mean_, ISOVF_max_, ISOVF_10th_, ISOVF_25th_, ISOVF_median_, ISOVF_75th_, ISOVF_90th_, ODI_mean_, ODI_max_, ODI_10th_, ODI_25th_, ODI_median_, ODI_75th_, ODI_90th_ and ODI_variance_ were significantly lower for atypical HGG than for PCNSL, whereas ADC_10th_, ADC_25th_, ADC_median_, ADC_minimum_, ADC_mean_, FA_10th_, FA_25th_, FA_median_, FA_75th_, FA_90th_, FA_minimum_, FA_mean_, ICVF_skewness_ and ODI_skewness_ were significantly higher for atypical HGG than for PCNSL (*p* < 0.05). The ISOVF_min_ values of atypical HGG and PCNSL were both < 0.001; therefore, no comparison was made.

**Table 1:** Histogram parameter values in the enhancing area.

Parameter	HGG	PCNSL	*t*/*U* value	Adjusted *p*
ADC_10th_	0.872 ± 0.162	0.712 ± 0.149	3.765^a^	<0.001^***^
ADC_25th_	0.946 ± 0.187	0.789 ± 0.138	3.501^a^	0.002^**^
ADC_median_	1.046 ± 0.218	0.905 ± 0.148	2.737^a^	0.013^*^
ADC_minimum_	0.721 (0.62, 0.823)	0.557 (0.437, 0.646)	604.5^c^	<0.001^***^
ADC_mean_	1.073 ± 0.216	0.958 ± 0.146	2.348^b^	0.031^*^
ADC_variance_	0.029 (0.02, 0.054)	0.049 (0.032, 0.068)	220^c^	0.013^*^
FA_10th_	0.103 (0.076, 0.164)	0.046 (0.036, 0.058)	688^c^	<0.001^***^
FA_25th_	0.139 (0.098, 0.205)	0.059 (0.046, 0.084)	689^c^	<0.001^***^
FA_median_	0.194 ± 0.075	0.095 ± 0.034	6.488^b^	<0.001^***^
FA_75th_	0.245 ± 0.092	0.15 ± 0.051	4.834^b^	<0.001^***^
FA_90th_	0.292 ± 0.107	0.227 ± 0.082	2.487^a^	0.023^*^
FA_minimum_	0.051 (0.029, 0.077)	0.016 (0.011, 0.022)	675^c^	<0.001^***^
FA_mean_	0.202 ± 0.076	0.12 ± 0.039	5.114^b^	<0.001^***^
FA_skewness_	0.55 (0.121, 0.863)	1.656 (1.404, 2.036)	74^c^	<0.001^***^
FA_kurtosis_	2.92 (2.532, 3.997)	5.523 (4.148, 7.874)	111^c^	<0.001^***^
ICVF_10th_	0.235 ± 0.088	0.304 ± 0.084	−2.944^a^	0.008^**^
ICVF_25th_	0.272 ± 0.097	0.393 ± 0.093	−4.729^a^	<0.001^***^
ICVF_median_	0.33 ± 0.105	0.487 ± 0.117	−5.232^a^	<0.001^***^
ICVF_75th_	0.358 (0.316, 0.437)	0.581 (0.467, 0.617)	108^c^	<0.001^***^
ICVF_90th_	0.437 ± 0.121	0.622 ± 0.144	−5.162^a^	<0.001^***^
ICVF_mean_	0.334 ± 0.099	0.474 ± 0.102	−5.149^a^	<0.001^***^
ICVF_max_	0.596 (0.463, 0.852)	0.93 (0.677, 0.986)	192^c^	0.003^**^
ICVF_variance_	0.006 (0.002, 0.013)	0.013 (0.008, 0.02)	191^c^	0.003^**^
ICVF_skewness_	0.484 (−0.065, 0.93)	−0.094 (−0.457, 0.213)	562^c^	0.003^**^
ISOVF_10th_	0.002 (0.001, 0.008)	0.017 (0.01, 0.038)	151^c^	<0.001^***^
ISOVF_25th_	0.011 (0.006, 0.044)	0.051 (0.041, 0.088)	163^c^	<0.001^***^
ISOVF_median_	0.047 (0.026, 0.104)	0.106 (0.086, 0.166)	190^c^	0.003^**^
ISOVF_75th_	0.135 (0.072, 0.207)	0.187 (0.153, 0.262)	217^c^	0.011^*^
ISOVF_90th_	0.259 (0.116, 0.315)	0.308 (0.243, 0.447)	232^c^	0.022^*^
ISOVF_mean_	0.1 (0.049, 0.148)	0.14 (0.112, 0.21)	213^c^	0.009^**^
ISOVF_max_	0.536 ± 0.232	0.726 ± 0.187	−3.3^a^	0.003^**^
ODI_10th_	0.236 ± 0.079	0.334 ± 0.107	−3.902^a^	<0.001^***^
ODI_25th_	0.297 ± 0.073	0.476 ± 0.126	−6.261^b^	<0.001^***^
ODI_median_	0.372 ± 0.081	0.646 ± 0.118	−9.824^b^	<0.001^***^
ODI_75th_	0.458 ± 0.092	0.768 ± 0.095	−12.221^a^	<0.001^***^
ODI_90th_	0.534 (0.445, 0.637)	0.859 (0.774, 0.882)	13^c^	<0.001^***^
ODI_mean_	0.382 ± 0.075	0.611 ± 0.098	−9.815^a^	<0.001^***^
ODI_max_	0.725 (0.658, 0.818)	0.933 (0.919, 0.946)	25^c^	<0.001^***^
ODI_variance_	0.013 (0.008, 0.019)	0.035 (0.027, 0.044)	49^c^	<0.001^***^
ODI_skewness_	0.356 ± 0.417	−0.592 ± 0.439	8.188^a^	<0.001^***^

a Data followed a normal distribution with homogeneity of variance; independent *t*-test was used.

b Data followed a normal distribution without homogeneity of variance; Welch’s *t*-test was used.

c Data did not follow a normal distribution; Mann–Whitney *U* test was used. ^*^0.01 < *p* ≤ 0.05; ^**^0.001 < *p* ≤ 0.01; ^***^p ≤ 0.001.

### 3.3 Histogram parameter values in peritumoral edema

**Figure 3:**
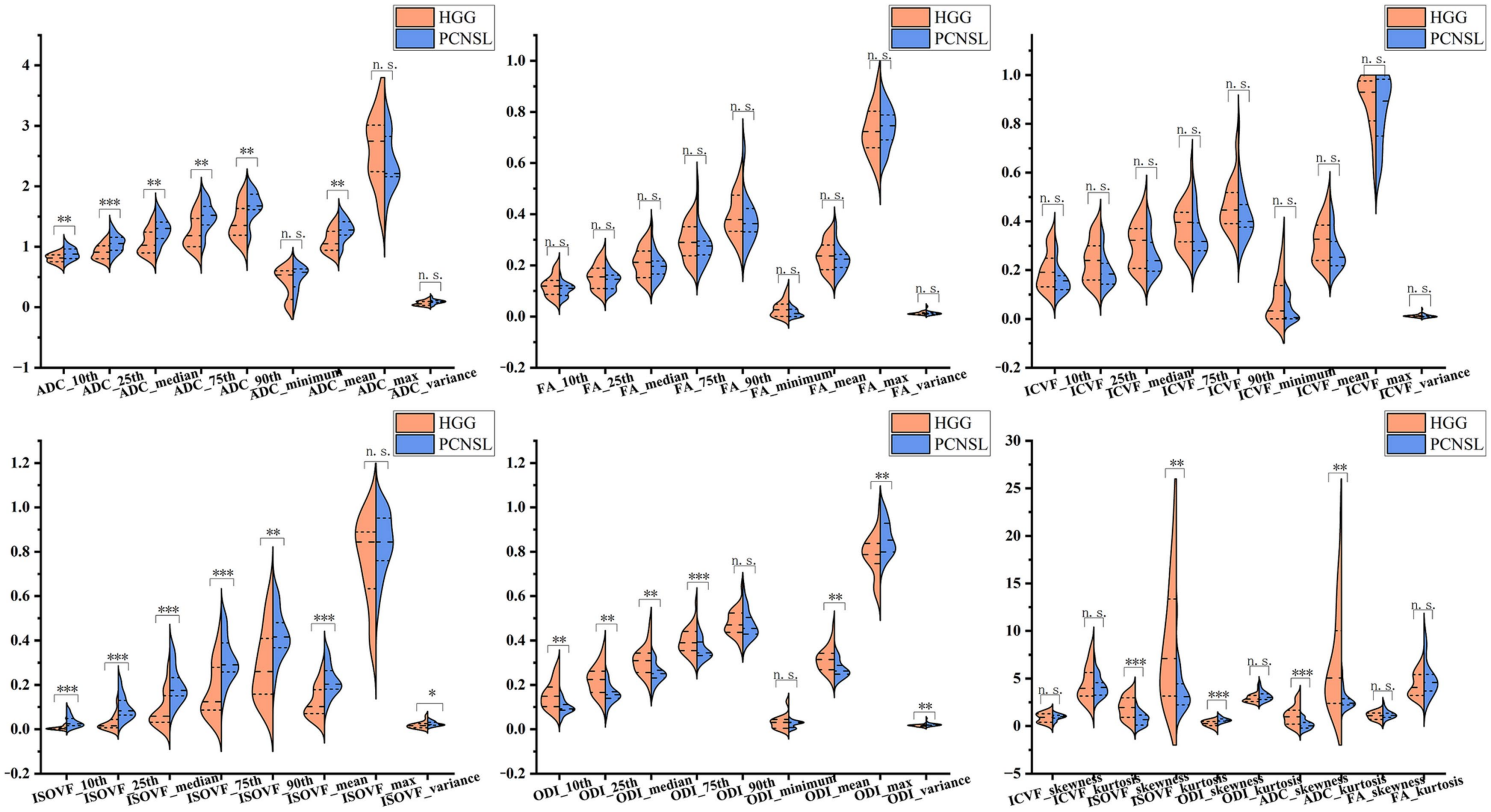
Violin graphs of histogram parameter values in the peritumoral edema. HGG, high-grade glioma; ADC, apparent diffusion coefficient; FA, fractional anisotropy; ICVF, intracellular volume fraction; ISOVF, isotropic volume fraction; ODI, orientation dispersion index; PCNSL, primary central nervous system lymphoma. ^*^0.01 < *p* ≤ 0.05; ^**^0.001 < *p* ≤ 0.01; ^***^p ≤ 0.001. n.s., no significance.

Table 2 and Figure 3 illustrate the histogram parameter values (ADC, FA, ICVF, ISOVF, and ODI) for peritumoral edema. In this area, the values of ADC_10th_, ADC_25th_, ADC_median_, ADC_75th_, ADC_90th_, ADC_mean_, ISOVF_mean_, ISOVF_10th_, ISOVF_25th_, ISOVF_median_, ISOVF_75th_, ISOVF_90th_, ISOVF_variance_, ODI_max_, ODI_variance_ and ODI_skewness_ were significantly lower for atypical HGG compared to PCNSL. Conversely, ADC_skewness_, ADC_kurtosis_, ISOVF_skewness_, ISOVF_kurtosis_, ODI_mean_, ODI_10th_, ODI_25th_ and ODI_median_, ODI_75th_ were significantly higher for atypical HGG compared to PCNSL (*p* < 0.05). As both the ISOVF_min_ of atypical HGG and PCNSL were < 0.001, a comparison was not conducted.

**Table 2:** Histogram parameter values in the peritumoral edema.

Parameter	HGG	PCNSL	*t*/*U* value	Adjusted *p*
ADC_10th_	0.808 ± 0.078	0.889 ± 0.096	−3.45^a^	0.003^**^
ADC_25th_	0.912 ± 0.122	1.056 ± 0.146	−3.996^a^	<0.001^***^
ADC_median_	1.027 (0.898, 1.238)	1.302 (1.139, 1.409)	170^c^	0.002^**^
ADC_75th_	1.183 (1.014, 1.462)	1.522 (1.361, 1.667)	181^c^	0.003^**^
ADC_90th_	1.353 (1.193, 1.631)	1.677 (1.615, 1.87)	182.5^c^	0.003^**^
ADC_mean_	1.096 ± 0.187	1.284 ± 0.175	−3.824^a^	0.002^**^
ADC_skewness_	0.968 (0.217, 1.645)	0.035 (−0.211, 0.382)	592^c^	<0.001^***^
ADC_kurtosis_	5.054 (2.563, 9.985)	2.405 (2.22, 2.884)	575^c^	0.002^**^
ISOVF_10th_	0.003 (0.001, 0.007)	0.025 (0.016, 0.049)	59^c^	<0.001^***^
ISOVF_25th_	0.016 (0.007, 0.042)	0.083 (0.064, 0.13)	75^c^	<0.001^***^
ISOVF_median_	0.059 (0.032, 0.136)	0.175 (0.15, 0.233)	116^c^	<0.001^***^
ISOVF_75th_	0.124 (0.088, 0.277)	0.291 (0.258, 0.389)	151^c^	<0.001^***^
ISOVF_90th_	0.29 ± 0.162	0.427 ± 0.108	−3.737^b^	0.002^**^
ISOVF_mean_	0.102 (0.07, 0.177)	0.203 (0.181, 0.264)	130^c^	<0.001^***^
ISOVF_variance_	0.016 (0.007, 0.025)	0.021 (0.018, 0.031)	239^c^	0.047^*^
ISOVF_skewness_	1.891 ± 1.137	0.753 ± 0.687	4.575^b^	<0.001^***^
ISOVF_kurtosis_	7.089 (3.258, 13.015)	3.105 (2.215, 4.453)	567^c^	0.003^**^
ODI_10th_	0.154 ± 0.062	0.097 ± 0.03	4.439^b^	<0.001^***^
ODI_25th_	0.223 ± 0.066	0.157 ± 0.044	4.436^b^	<0.001^***^
ODI_median_	0.308 ± 0.064	0.251 ± 0.045	3.748^a^	0.002^**^
ODI_75th_	0.39 (0.355, 0.439)	0.344 (0.332, 0.393)	535^c^	0.016^*^
ODI_mean_	0.314 ± 0.058	0.27 ± 0.038	3.247^a^	0.005^**^
ODI_max_	0.781 ± 0.094	0.86 ± 0.077	−3.397^a^	0.003^**^
ODI_variance_	0.017 (0.013, 0.019)	0.019 (0.018, 0.023)	182^c^	0.003^**^
ODI_skewness_	0.257 ± 0.336	0.656 ± 0.245	−4.934^a^	<0.001^***^

a Data followed a normal distribution with homogeneity of variance; independent *t*-test was used.

b Data followed a normal distribution without homogeneity of variance; Welch’s *t*-test was used.

c Data did not follow a normal distribution; Mann–Whitney *U* test was used. ^*^0.01 < *p* ≤ 0.05; ^**^0.001 < *p* ≤ 0.01; ^***^p ≤ 0.001.

### 3.4 Performance of histogram parameters from both ROIs in differentiating atypical HGG from PCNSL

**Figure 4:**
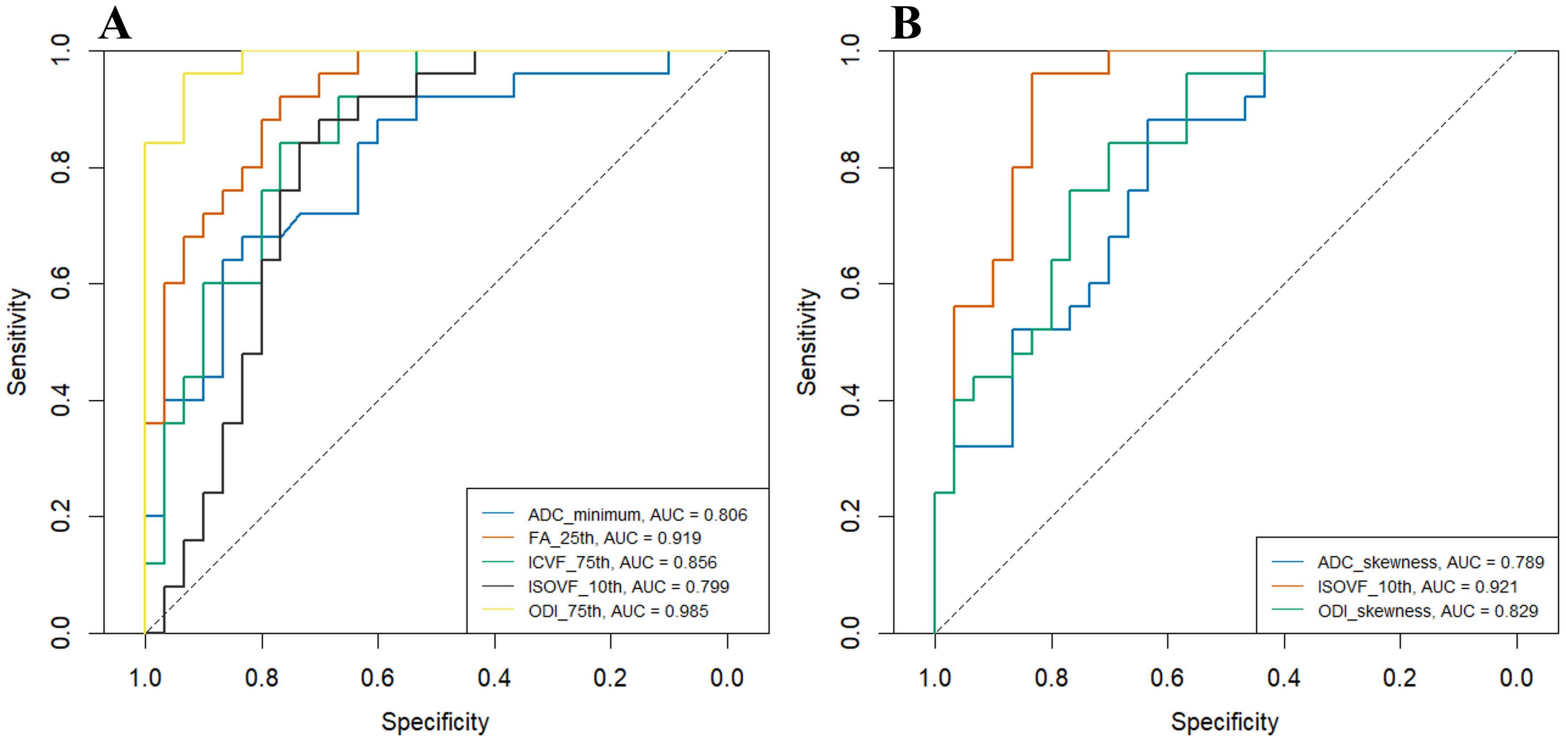
The ROC curves for histogram parameter values from the enhancing area **(A)** and the peritumoral edema **(B)** in distinguishing between atypical HGG and PCNSL. HGG, high-grade glioma; ADC, apparent diffusion coefficient; FA, fractional anisotropy; ICVF, intracellular volume fraction; ISOVF, isotropic volume fraction; ODI, orientation dispersion index; PCNSL, primary central nervous system lymphoma.

Table 3 presents the ROC analyses of all significant histogram parameter values in the enhancing areas. Figures 4A,B present the ROC curves of the histogram parameters with the highest AUC from each model in the enhancing and peritumoral edema regions, respectively. Notably, the ODI_75th_ displayed the highest AUC of 0.985 [95% confidence interval (CI): 0.957–1.000] with an accuracy of 0.945 (52/55) at the optimal threshold of 0.604. Its corresponding sensitivity and specificity were 0.96 and 0.933, respectively. The AUC of the other parameters ranged from 0.655–0.983.

**Table 3:** ROC analyses of significant histogram parameters in the enhancing area.

Parameter	AUC (95% CI)	Cut-off value	Sensitivity	Specificity	Accuracy
ADC_10th_	0.773 (0.64, 0.889)	0.8	0.880	0.667	0.764
ADC_25th_	0.755 (0.619, 0.872)	0.868	0.840	0.667	0.745
ADC_median_	0.703 (0.561, 0.836)	0.923	0.680	0.733	0.709
ADC_minimum_	0.806 (0.683, 0.911)	0.607	0.680	0.833	0.764
ADC_mean_	0.655 (0.505, 0.803)	1.072	0.880	0.467	0.655
ADC_variance_	0.707 (0.561, 0.832)	0.03	0.800	0.600	0.691
FA_10th_	0.917 (0.833, 0.979)	0.069	0.920	0.800	0.855
FA_25th_	0.919 (0.84, 0.975)	0.096	0.920	0.767	0.836
FA_median_	0.9 (0.804, 0.964)	0.125	0.840	0.800	0.818
FA_75th_	0.812 (0.691, 0.917)	0.179	0.760	0.767	0.764
FA_90th_	0.684 (0.541, 0.821)	0.214	0.560	0.767	0.673
FA_minimum_	0.9 (0.811, 0.968)	0.025	0.800	0.833	0.818
FA_mean_	0.829 (0.711, 0.925)	0.147	0.800	0.733	0.764
FA_skewness_	0.901 (0.809, 0.971)	1.005	0.920	0.833	0.873
FA_kurtosis_	0.852 (0.736, 0.943)	3.218	1.000	0.667	0.818
ICVF_10th_	0.715 (0.573, 0.845)	0.239	0.800	0.600	0.691
ICVF_25th_	0.815 (0.691, 0.913)	0.296	0.840	0.700	0.764
ICVF_median_	0.855 (0.751, 0.943)	0.337	0.920	0.667	0.782
ICVF_75th_	0.856 (0.748, 0.94)	0.439	0.840	0.767	0.800
ICVF_90th_	0.849 (0.747, 0.94)	0.465	0.920	0.700	0.800
ICVF_mean_	0.847 (0.732, 0.936)	0.356	0.920	0.667	0.782
ICVF_max_	0.744 (0.611, 0.865)	0.493	1.000	0.400	0.673
ICVF_variance_	0.745 (0.609, 0.859)	0.006	0.920	0.533	0.709
ICVF_skewness_	0.749 (0.619, 0.867)	0.428	0.920	0.533	0.709
ISOVF_10th_	0.799 (0.663, 0.913)	0.005	0.880	0.700	0.782
ISOVF_25th_	0.783 (0.653, 0.9)	0.022	0.880	0.700	0.782
ISOVF_median_	0.747 (0.601, 0.871)	0.055	0.960	0.567	0.745
ISOVF_75th_	0.711 (0.567, 0.839)	0.122	0.960	0.500	0.709
ISOVF_90th_	0.691 (0.549, 0.82)	0.165	1.000	0.367	0.655
ISOVF_mean_	0.716 (0.572, 0.841)	0.084	0.960	0.467	0.691
ISOVF_max_	0.733 (0.593, 0.857)	0.601	0.760	0.667	0.709
ODI_10th_	0.783 (0.652, 0.897)	0.3	0.600	0.867	0.745
ODI_25th_	0.897 (0.796, 0.972)	0.333	0.920	0.733	0.818
ODI_median_	0.969 (0.927, 0.996)	0.438	1.000	0.800	0.891
ODI_75th_	0.985 (0.957, 1)	0.604	0.960	0.933	0.945
ODI_90th_	0.983 (0.952, 1)	0.687	0.960	0.933	0.945
ODI_mean_	0.961 (0.908, 0.995)	0.451	0.960	0.833	0.891
ODI_max_	0.967 (0.919, 0.995)	0.86	1.000	0.833	0.909
ODI_variance_	0.935 (0.861, 0.992)	0.024	0.960	0.867	0.909
ODI_skewness_	0.957 (0.899, 0.995)	−0.059	0.880	0.933	0.909

Table 4 display the ROC analyses of all significant histogram parameter values for peritumoral edema. In this context, the ISOVF_10th_ exhibited the highest AUC of 0.921 (95% CI: 0.839–0.98) with an accuracy of 0.891 (49/55) at an optimal threshold of 0.009. The corresponding sensitivity and specificity were 0.96 and 0.833, respectively. The AUCs for the remaining parameters ranged from 0.681–0.900.

**Table 4:** ROC analyses of significant histogram parameters in the peritumoral edema.

Parameter	AUC (95% CI)	Cut-off value	Sensitivity	Specificity	Accuracy
ADC_10th_	0.734 (0.594, 0.859)	0.867	0.600	0.767	0.691
ADC_25th_	0.778 (0.647, 0.892)	1.04	0.600	0.867	0.745
ADC_median_	0.773 (0.639, 0.885)	1.09	0.880	0.600	0.727
ADC_75th_	0.759 (0.623, 0.884)	1.288	0.880	0.633	0.745
ADC_90th_	0.757 (0.625, 0.884)	1.45	0.880	0.633	0.745
ADC_mean_	0.765 (0.629, 0.883)	1.122	0.880	0.600	0.727
ADC_skewness_	0.789 (0.661, 0.892)	0.851	0.880	0.633	0.745
ADC_kurtosis_	0.767 (0.633, 0.887)	3.478	0.840	0.667	0.745
ISOVF_10th_	0.921 (0.839, 0.98)	0.009	0.960	0.833	0.891
ISOVF_25th_	0.9 (0.809, 0.971)	0.024	1.000	0.700	0.836
ISOVF_median_	0.845 (0.737, 0.939)	0.092	0.960	0.733	0.836
ISOVF_75th_	0.799 (0.672, 0.901)	0.227	0.880	0.700	0.782
ISOVF_90th_	0.764 (0.629, 0.892)	0.328	0.880	0.633	0.745
ISOVF_mean_	0.827 (0.705, 0.925)	0.148	0.880	0.667	0.764
ISOVF_variance_	0.681 (0.535, 0.815)	0.018	0.800	0.567	0.673
ISOVF_skewness_	0.791 (0.66, 0.897)	1.78	0.960	0.567	0.745
ISOVF_kurtosis_	0.756 (0.617, 0.879)	6.065	0.880	0.633	0.745
ODI_10th_	0.796 (0.663, 0.908)	0.127	0.880	0.633	0.745
ODI_25th_	0.799 (0.669, 0.911)	0.199	0.840	0.667	0.745
ODI_median_	0.771 (0.644, 0.887)	0.271	0.800	0.700	0.745
ODI_75th_	0.713 (0.575, 0.847)	0.347	0.640	0.800	0.727
ODI_mean_	0.745 (0.604, 0.868)	0.268	0.640	0.800	0.727
ODI_max_	0.743 (0.605, 0.867)	0.787	0.920	0.533	0.709
ODI_variance_	0.757 (0.62, 0.879)	0.019	0.680	0.733	0.709
ODI_skewness_	0.829 (0.708, 0.921)	0.464	0.840	0.700	0.764

## 4 Discussion

In this study, we evaluated the diagnostic value of DTI-and NODDI-based histogram analysis in distinguishing between atypical HGG and PCNSL. Multiple histogram parameters in the enhancing area or peritumoral edema were shown to be effective in this discrimination, and the ODI_75th_ from the enhancing area presented the strongest diagnostic capability.

Histogram-based features have demonstrated high applicability in characterizing highly heterogeneous tumor tissues (24). Compared to conventional summary metrics such as the mean or median, ODI_75th_ and ODI_90th_ provide more targeted information about regions where white matter fiber tracts are substantially disrupted by tumor infiltration. Unlike extreme values (e.g., maximum or minimum), close to percentiles are less susceptible to noise and contamination caused by errors in VOI delineation or image registration (15, 25), notably, among the histogram-derived parameters of FA, FA_25th_ and FA_90th_ exhibited the highest AUC values. Given the mathematically inverse relationship between FA and ODI (13), parameters such as FA_25th/10th_ and ODI_75th/90th_ may reflect similar tumor subregions—specifically, regions within the tumor parenchyma where axonal fibers demonstrate pronounced alterations, including crossing, curving, or disorganization. Alterations in intra-tumoral neural fiber tracts are difficult to observe with conventional MRI but can be detected using advanced diffusion magnetic resonance techniques, which can subsequently be employed to characterize the tumor’s growth behavior. ODI reflects the spread of neurite orientation. It can portray the microstructural complexity, particularly the bending of white matter axons and the pattern of gray matter dendrite expansion (13, 26, 27). We found ODI values are lower and FA values are higher in atypical HGG than in PCNSL. Previous study also indicated significantly higher FA in HGG compared to PCNSL (28), which is attributed to the disparity in the quantity of nerve fibers between the two tumors. However, explaining the parameter variations solely by the difference in neurite density might lead to a conflict between the two parameters, ODI and ICVF. In our study, atypical HGG displayed conspicuously lower ICVF values in contrast to PCNSL. Higher ICVF skewness in atypical HGG complementarily indicates the trend of lower ICVF values distribution in HGG. As per the definition of the NODDI model, water diffusion within voxels is partitioned into separate contributions from three compartments, with ICVF being the fraction of intracellular volume within voxels, representing neurite density (27). This variance might be ascribed to the presence of microcysts in HGG (30) and the relatively high neurite density in PCNSL (31). Würtemberger et al. (14) confirmed the higher axonal density in PCNSL with histological evidence. Considering our results and evidence presented in these studies, we propose that while more axonal structures are preserved within PCNSL, the tumor’s expansive growth displaces, compresses, and intersects with fibrous structures, leading to a decrease in isotropy. In HGG, due to its invasive growth pattern, extensive fiber destruction occurs, leaving the remaining fibrous structures with greater directionality. Consequently, ODI is lower in HGG. In the study by Würtemberger et al. (14), a similar trend in ODI was observed, but without statistical significance, likely due to the limited sample size. ISOVF represents an isotropic diffusion signal from the cerebrospinal fluid compartment, and deviations in ISOVF values suggest a higher content of free water in PCNSL tissues. However, these findings are inconsistent with those reported by Würtemberger et al. (14). In both our study and theirs, the role of ISOVF in differential diagnosis appears limited, as evidenced by its relatively low AUC value. This suggests that the diagnostic utility of ISOVF remains uncertain and warrants further validation in studies with larger sample sizes. Lower ADC values in PCNSL suggest higher cellularity compared to HGG, which has been histologically confirmed in Haopeng et al. (29) study.

ODI demonstrated superior diagnostic performance compared to FA, largely owing to the NODDI model’s ability to more specifically represent white matter fiber architecture (13). FA is influenced by a variety of factors, including fiber orientation coherence and neurite density. In PCNSL, the disruption and displacement of white matter tracts typically result in a reduction of FA, while high neurite density can increase FA. These opposing effects may partially cancel each other out, thereby reducing the discriminatory power of FA in differentiating PCNSL from HGG. In contrast, the NODDI model decomposes these microstructural changes into distinct parameters: ICVF reflects neurite density, and ODI quantifies the orientation dispersion of fibers. By isolating orientation dispersion from confounding factors such as cellular density, ODI provides a more robust metric that more accurately reflects the architectural disorganization characteristic of PCNSL, yielding a clearer distinction from HGG.

However, the biological interpretation of NODDI-derived parameters in tumor tissue remains largely empirical and requires further histological validation. This model was originally designed to characterize white matter microstructure in healthy or neurodegenerative brains—such as in neonates or patients with dementia (32)—and lacks explicit modeling for neoplastic components. Neoplastic cells may exhibit restricted diffusion similar to that of neurites, and thus may be erroneously classified into the intracellular compartment, resulting in an artificial elevation of ICVF. This misclassification could misleadingly suggest preserved neuronal integrity in regions actually dominated by tumor cells. Similarly, the disorganized microstructure introduced by malignant proliferation may be interpreted as increased neurite dispersion, contributing to inflated ODI values. Moreover, the NODDI model does not account for perfusion effects, which are commonly observed in highly vascularized tumors. This becomes particularly problematic at lower b-values: in our protocol, the inclusion of a *b* = 500 s/mm^2^ shell may have introduced mild perfusion contamination. Fast diffusion components retained at this b-value may be misclassified as extracellular space, potentially leading to overestimation of ISOVF and underestimation of ICVF. Also, ODI may become unstable due to contamination by randomly flowing blood signals. Nevertheless, the clinical potential of the NODDI model in tumor diagnostics has been supported by a growing body of research. Quantitative NODDI parameters have been applied in glioma grading and molecular subtype prediction (33, 34), NODDI-based tractography has proven effective in evaluating corticospinal tract (CST) infiltration and damage caused by HGGs (35). Furthermore, multiparametric NODDI radiomic models have demonstrated good performance in the preoperative differentiation of glioblastoma and brain metastases (36). Compared to NODDI, several other diffusion models may offer better theoretical alignment and practical utility for tumor microstructure characterization with their assumptions that are more appropriate for pathological tissues. The vascular, extracellular, and restricted diffusion for cytometry in tumors (VERDICT) model explicitly incorporates perfusion and distinguishes between key microenvironmental components (37), providing estimates for intracellular volume fraction (fIC), vascular volume fraction (fVASC), extracellular-extravascular space (fEES), and cell radius. This enables a more comprehensive representation of tumor cellularity and vascularization. The imaging microstructural parameters using limited spectrally edited diffusion (IMPULSED) model is capable of quantifying cell membrane permeability (38), a parameter increasingly recognized for its value in assessing tumor viability and treatment response. Single-compartment models such as diffusion kurtosis imaging (DKI) and mean apparent propagator MRI (MAP-MRI) yield metrics that reflect tissue heterogeneity, and have shown promise in tumor subtype classification and prediction of molecular phenotypes (34, 39).

NODDI could be more appropriate for characterizing the peritumoral edema region, where the predominant constituents are edematous brain parenchyma and infiltrated white matter tracts rather than compact tumor cells. In these regions, the diffusion environment still partially conforms to the assumptions of the NODDI framework ISOVF demonstrated a unique diagnostic value in the peritumoral edema region. Although it did not exhibit the highest diagnostic performance in this study, it holds merit for elucidating the underlying pathophysiological processes and characterizing the microstructural differences in the edema regions of HGG and PCNSL. Its differential diagnostic potential is likely to be further revealed in future large-scale studies that establish multifactorial models. We noticed significantly lower values of ISOVF, higher ISOVF skewness and ISOVF kurtosis in atypical HGG compared to PCNSL. This disparity might be ascribed to the infiltrative edema characteristic of HGG (40, 41), which comprises infiltrating tumor cells and vasogenic edema. Vasogenic edema mainly results from the tumor’s pressure on draining veins, without the presence of tumor cells within the edema (42). In contrast to the ‘pure vasogenic edema’ (with no infiltrating tumor cells) observed around PCNSL (43), in the peritumoral area of HGG, the infiltrating tumor cells reduce the free water content. ISOVF_10th_, the parameter with the highest diagnostic performance in the edema region (AUC = 0.921), corresponds to areas with relatively low free water content. This effectively reflects the invasive growth behavior of tumor cells in HGG, highlighting its potential as a valuable biomarker for characterizing tumor-related microstructural changes. In the peritumoral edema of atypical HGG, infiltrating tumor cells disrupt nerve fibers, leading to more dispersed water diffusion. This results in higher values for ODI_mean_, ODI_10th_, ODI_25th_, ODI_median_, and ODI_75th_ in atypical HGG compared to PCNSL. Interestingly, however, ODI_max_ was found to be higher in the edema surrounding PCNSL. This may reflect the characteristics of edematous brain tissue at the tumor-edema interface, where fiber tracts are maximally displaced or intersecting, possibly due to the expansile growth pattern of PCNSL. Such regions may harbor sharply distorted or crossing fibers compressed by the tumor mass rather than infiltrated by tumor cells. Despite this localized increase in ODI_max_, the overall distribution of ODI values is shifted higher in the peritumoral region of HGG, as supported by differences in skewness. These findings underscore the potential of ODI metrics to capture the infiltrative growth behavior of HGG, in contrast to the vasogenic edema in PCNSL, which primarily results from blood–brain barrier disruption with limited tumor infiltration.

This study has several limitations. First, it was conducted at a single center using a single scanner and involved a relatively small sample size. Future studies with larger cohorts, including data from multiple scanners and institutions, are needed to validate these findings. Second, the lack of a validation cohort prevented the development of a multifactorial diagnostic model that integrates parameters from both the edema and enhancement areas. We plan to address this limitation by enrolling more patients in subsequent studies. Third, the interpretation of diffusion parameter changes in this study is empirical and lacks histopathological validation, highlighting the need for future studies incorporating histological correlation.

## 5 Conclusion

DTI-and NODDI-based histogram analysis shows promise in distinguishing between atypical HGG and PCNSL, with the ODI_75th_ parameter from the enhancing area demonstrating the best diagnostic performance.

## Data Availability

The raw data supporting the conclusions of this article will be made available by the authors, without undue reservation.

## Footnotes

^1^ https://diffusionkit.readthedocs.io

^2^ http://nipy.org/
